# Outcome Measures Following Critical Illness in Children With Disabilities: A Scoping Review

**DOI:** 10.3389/fped.2021.689485

**Published:** 2021-07-02

**Authors:** Julia A. Heneghan, Sarah A. Sobotka, Madhura Hallman, Neethi Pinto, Elizabeth Y. Killien, Kathryn Palumbo, Sinead Murphy Salem, Kilby Mann, Barbara Smith, Rebecca Steuart, Manzilat Akande, Robert J. Graham

**Affiliations:** ^1^Division of Pediatric Critical Care Medicine, University of Minnesota Masonic Children's Hospital, Minneapolis, MN, United States; ^2^Section of Developmental and Behavioral Pediatrics, Department of Pediatrics, The University of Chicago, Chicago, IL, United States; ^3^Division of Pediatric Critical Care Medicine, University of Alabama at Birmingham, Birmingham, AL, United States; ^4^Department of Anesthesiology and Critical Care Medicine, Children's Hospital of Philadelphia, Philadelphia, PA, United States; ^5^Division of Pediatric Critical Care Medicine, Department of Pediatrics, Seattle Children's Hospital, University of Washington School of Medicine, Seattle, WA, United States; ^6^Department of Pediatrics, University of Rochester Medical Center, Rochester, NY, United States; ^7^Department of Anesthesiology, Critical Care, and Pain Medicine, Boston Children's Hospital, Boston, MA, United States; ^8^Department of Physical Medicine and Rehabilitation, Children's Hospital Colorado, University of Colorado, Aurora, CO, United States; ^9^Departments of Physical Therapy and Pediatrics, University of Florida, Gainesville, FL, United States; ^10^Division of Hospital Medicine, Cincinnati Children's Medical Center, Cincinnati, OH, United States; ^11^Department of Pediatrics, Oklahoma University Health Sciences Center, Oklahoma, OK, United States

**Keywords:** intensive care, pediatrics, disabilities, children with medical complexity, outcomes

## Abstract

Children with disabilities compose a substantial portion of admissions and bed-days in the pediatric intensive care unit (PICU) and often experience readmissions over time. Impacts of a PICU admission on post-discharge health status may be difficult to distinguish from pre-existing disability in this population. Efforts to standardize outcome measures used for children with disabilities may help identify morbidities associated with PICU hospitalizations. Although a scoping review of outcome measures to assess children after episodes of critical illness has recently been published, it is not known to what extent these measures are appropriate for use in children with disabilities. This limits our ability to effectively measure long-term outcomes following critical illness in this important patient population. Through mixed methodology of scoping review and multi-stakeholder consensus, we aimed to identify and describe instruments previously utilized for this purpose and to explore additional tools for consideration. This yielded 51 measures across a variety of domains that have been utilized in the PICU setting and may be appropriate for use in children with disabilities. We describe characteristics of these instruments, including the type of developmental domains assessed, availability of population data, validation and considerations regarding administration in children with disabilities, and ease of availability of the instrument to researchers. Additionally, we suggest needed alterations or accommodations for these instruments to augment their utility in these populations, and highlight areas for future instrument development.

## Introduction

Episodes of pediatric critical illness may result in mortality or morbidity across a range of domains in a child's functioning. A growing focus among practitioners of pediatric critical care is post-intensive care syndrome-pediatrics (PICS-p), which is characterized by potential changes in multiple domains of functioning in survivors of pediatric critical illness, including neurocognition, physical functioning, social functioning, and health-related quality of life (1). However, many children hospitalized with critical illness have baseline developmental delays and disabilities (2–5). These patients have been shown to be at increased risk for critical illness, as well as for death, prolonged intensive care unit length of stay, and higher medical resource utilization during episodes of critical illness (5–9). In addition, many children with complex medical conditions may be at higher risk for impaired outcomes due to their underlying diseases, susceptibility to adverse effects of therapeutic interventions, or missed educational and therapy experiences while hospitalized, making them among the most vulnerable of pediatric intensive care unit (PICU) patients.

Recent work has resulted in a scoping review as well as a core set of outcome measures used to assess children after episodes of critical illness with a focus on these PICS-p domains (10, 11). However, it is not known whether these measures are appropriate for use in children with pre-existing disabilities or complex medical needs, who make up a substantial portion of admissions to the modern PICU (5, 8, 9). Definitions and categorization of medical complexity [e.g., Children with Special Healthcare Needs (12, 13), Complex Chronic Conditions (14), Pediatric Medical Complexity Algorithm (15), Pediatric Chronic Critical Illness (16)] differ, may inconsistently overlap (17, 18), and variably incorporate assessments of functional status. Notably, not all children with medical complexity will have disabilities, and vice versa. This heterogeneity in definitions highlights the need for identifying or developing a range of instruments to capture meaningful and patient-centered outcomes, taking into consideration individual patient baselines, which may differ from population or age-based normal values. We aimed to identify and describe characteristics of instruments that may be useful to all stakeholders, including but not limited to families, intensivists, and continuity providers (e.g., medical, educational, and therapy-based professionals) in the longitudinal evaluation of children with disabilities following critical illness.

## Materials and Methods

The Pediatric Acute Lung Injury and Sepsis Investigators (PALISI) network POST-PICU Investigators and the *Eunice Kennedy Shriver* National Institute for Child Health and Human Development Collaborative Pediatric Critical Care Research Network (CPCCRN) conducted a scoping review of all non-mortality outcomes measured following pediatric critical illness to inform the development of a core outcome set for use in pediatric critical care outcomes research. Details of the scoping review (9) and the core outcome set (10) have been previously published. In brief, PubMed, EMBASE, PsycINFO, Cumulative Index of Nursing and Allied Health Literature, and the Cochrane Central Register of Controlled Trials Registry were queried to identify studies published between 1970 and 2017 evaluating the outcomes of survivors or families after pediatric critical illness. Studies were excluded if no post-discharge outcomes were assessed or if mortality was the only outcome examined; if the included patients were primarily adults (>18 years), preterm infants, or neonates; if the patient had not been definitively admitted to an ICU or there was no clear relationship to ICU care (e.g., only a technical procedure/condition was evaluated); if only a single subject was included; if only psychometric properties of an instrument were evaluated or reported; or if the study was not available in English. Each manuscript was dual reviewed for eligibility and each potentially eligible manuscript was subsequently dual screened for final eligibility, with discrepancies resolved by a third reviewer. Information from each manuscript, including study characteristics, was then separately extracted by two reviewers, with discrepancies resolved through consensus. This scoping review identified 366 unique instruments. Of these, 136 were selected for further review by identifying the five most commonly used instruments in each domain, as well as any instrument used in publications from 2007 to 2017.

For the purposes of this study, we included instruments from the prior review where the investigators had indicated use in children with disabilities in order to capture a population likely to overlap with the general PICU population. This yielded 49 instruments. This list was then further narrowed to include only instruments that were used more than once in the scoping review literature base. Instruments were then reviewed by content-area experts (SS, developmental and behavioral pediatrics; KM, pediatric physiatry; BS, pediatric physical therapy) and additional instruments commonly used by experts to assess diverse abilities and disabilities as well instruments which came into use following the conclusion of the scoping review were added. This resulted in a final list of 51 instruments. Additional focused data collection was undertaken to assess how the instruments were used and applied to children with disabilities and to confirm the validity of previously collected data. We abstracted data on instrument characteristics (e.g., suggested age range, reported method and duration of administration, cost, training needed for administration), available information regarding population data for children with disabilities, the types of functioning assessed, and publisher information. The Preferred Reporting Items for Systematic Reviews and Meta-Analysis Extension for Scoping Reviews (PRISMA-ScR) Checklist was followed (Supplementary Material).

Instruments were classified into nine categories [cognitive functioning, executive functioning, communication, physical functioning, social skills, feeding, family functioning and child quality of life, mental health (e.g., anxiety, depression, trauma), and sleep] in order to capture and delineate complex neurodevelopmental outcomes and align with typical neurodevelopmental domains. These differed from the four categories outlined in PICS-p (Physical Health, Cognitive Health, Emotional Health, and Social Health) in order to best capture the intention of the measures designed and terminology used, but physical health likely relates to our physical functioning domain, cognitive health likely includes both cognitive functioning and executive functioning, emotional health likely includes mental health, and social health likely includes family functioning and child quality of life. The additional domains (communication, social skills, feeding, and sleep) were added to build upon the existing PICS-p framework and reflect developmental domains previously tested by studies evaluating post-PICU outcomes.

Study data for both the overall scoping review and this project were collected and managed using Research Electronic Data Capture (REDCap) hosted, respectively, at the University of Utah and the University of Minnesota. Included data are presented as frequency for categorical data and median and interquartile range (IQR) for continuous data. Data analysis was performed in R (R Foundation for Statistical Computing; Vienna, Austria).

## Results

**Figure 1:**
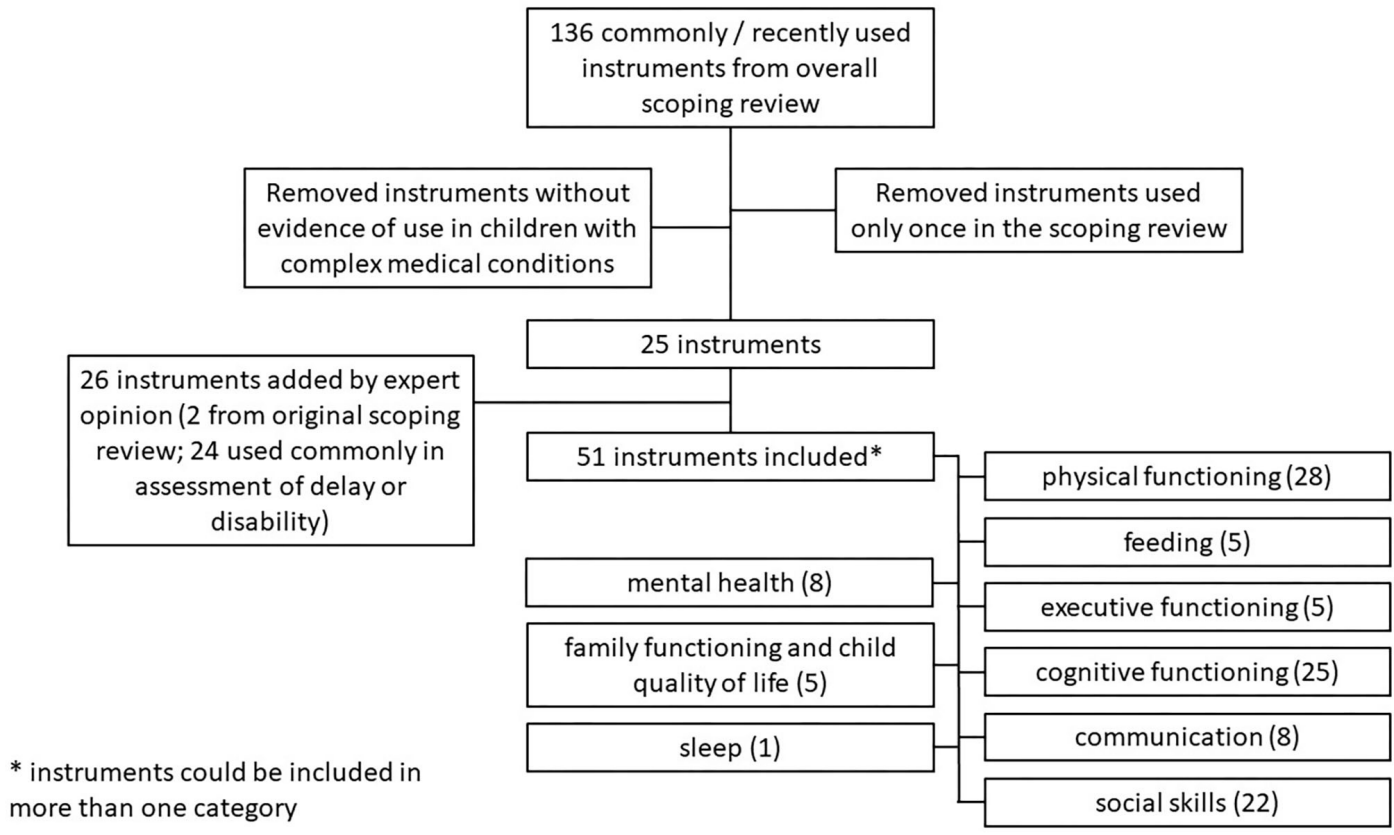
Flow diagram of included instruments by category.

One hundred thirty-six instruments were identified as being commonly or recently used to measure post-discharge outcomes after PICU care as part of the larger scoping review (10). Of these, 49 (36.0%) instruments were identified as having been used in children with disabilities. Of the 51 instruments ultimately included in this study, 27 (52.9%) were drawn from the primary scoping review; 2 (3.9%) were initially excluded based on only a single use in the scoping review but added back based upon expert opinion. The remaining 24 (47.1%) were included based on expert opinion alone. A flow diagram of instruments is shown in Figure 1. PICS-p domains of focus did not vary significantly between those instruments identified in the scoping review and those identified by expert opinion (data not shown). A list of all included instruments divided by domain of functioning assessed, as well as selected characteristics of each instrument can be found in Tables 1–9.

**Table 1:** Cognitive functioning.

**Instrument name**	**Additional domains**	**Suggested age range**	**Data source**	**Training for administration**	**Time and method of administration**	**Cost information**	**Website/additional info**
A Developmental Neuropsychological Assessment (NEPSY)^c^^,^ ^s^	Executive Functioning, Communication	3–16 years	Self-report, clinician	Instrument-specific training	45–180 min, in person	Initial kit: $1,023	Pearson Assessments: NEPSY
Adaptive Behaviour Assessment System (ABAS)^p^^,^ ^c^	Physical, Social, Communication	Up to 89 years	Self-report, parent or caregiver, teacher	Instrument-specific training	20 min, in person or electronic	Initial kit: $456	Pearson Assessments: ABAS
Ages and Stages Questionnaires^^p^^,^^c^^,^^s^^	Physical, Social, Communication	1 month−5.5 years	Parent or caregiver	None	10–15 min, in person	Initial kit: $295	Ages & Stages
Amsterdam Neuropsychological Tasks^c^^,^ ^s^	Social	4–65 years	Self-report	None	315–415 min, electronic	1 year license: €1600	ANT Program
*Bayley Scales of Infant and Toddler Development-4*^p^^,^^c^^,^^s^**	*Physical, Social, Communication*	*16 days−3.5 years*	*Clinician*	*Instrument-specific training*	*30*–*70 min, in person*	*Initial kit: $1169*	*Pearson Assessments: Bayley*
Cambridge Neuropsychological Test Automated Battery (CANTAB)^c^^,^ ^s^	Executive Function, Social	At least 4 years	Self-report	Instrument-specific training	Depends on modules selected (usually >30 min) electronic	Fee not available	CANTAB
Child Health Questionnaire (CHQ)^p, c, e, s^	Physical, Social, Family	5–18 years	Self-report, parent or caregiver	None	5–15 min; in person, *via* mail, or electronic	Fee not available	CHQ
*Caregiver Priorities and Child Health Index of Life with Disabilities (CPCHILD)^*p, c, e, s*^*	*Physical, Social, Family*	*5–18 years*	*Parent or caregiver*	*None*	*20*–*40 min, in person*	*Free*	*CPCHILD*
Denver Developmental Screening Test II^^p^^,^^c^^,^^s^^	Physical, Social	Up to 6 years	Clinician	Instrument-specific training	10–30 min, in person	Fee not available	Denver II
*Developmental Profile 3 (DP3)*^p^^,^^c^^,^^s^**	*Physical, Social*	*Up to 13 years*	*Self-report, parent or caregiver*	*Instrument-specific training*	*20*–*40 min, in person or by phone*	*Initial kit: $125*	*DP3*
Functional Independence Measures (FIM, WeeFIM)^p^^,^ ^c^	Physical, Feeding	6 months−21 years	Self-report, parent or caregiver	Instrument-specific training	15 min; in person, *via* mail, or by phone	Licensing: $2,200–4,100	FIM/WeeFIM
Functional Status II-R (FSII-R)^^p^^,^^c^^,^^s^^	Physical, Social, Feeding	Up to 16 years	Parent or caregiver	None	15 min, in person	Free	–
Functional Status Scale (FSS)^p^^,^ ^c^	Physical, Feeding	Up to 18 years	Clinician	None	<5 min, in person or chart review	Free	FSS
*Glasgow Outcome Scale-Pediatrics (GOSE-Peds)^*p, c, e, s*^*	*Physical, Social, Family*	*Up to 16 years*	*Self-report, parent or caregiver, clinician*	*None*	*15 min, in person or via phone*	*Free*	–
Heath State Utility Index (HUI)^^p^^,^^c^^,^^s^^	Physical, Social	5–100 years	Self-report, parent or caregiver, clinician, family member	Instrument-specific training	3–10 min; in person, by phone, or electronic	Free for information available in the literature, additional licensing: $5,000	HUI
King's Outcome Scale for Childhood Head Injury (KOSCHI)^^p^^,^^c^^,^^s^^	Physical, Social	Up to 16 years	Clinician	None	Variable timing, chart review	Free	KOSCHI
*Pediatric Cerebral Performance Category (PCPC)^*c*^*	–	*Up to 18 years*	*Parent or caregiver, clinician*	*None*	*5*–*10 min, in person or chart review*	*Free*	–
Pediatric Evaluation of Disability Inventory - Computer Adaptive Test (PEDICAT)^^p^^,^^c^^,^^s^^	Physical, Social, Feeding	Up to 20 years	Self-report, parent or caregiver, clinician	None	10–30 min, electronic	$2 per administration	Pearson Assessments: PEDICAT
Pediatric Overall Performance Category (POPC)^p^^,^ ^c^	Physical	Up to 21 years	Parent or caregiver, clinician	None	5–10 min, in person or chart review	Free	–
Pediatric Quality of Life Inventory (PedsQL)^p, c, e, s^	Physical, Social, Family	1 month−25 years	Self-report, parent or caregiver	None	4 min; in person, by phone, or *via* mail	Free for unfunded research; $1,089 for funded research	PEDSQL
Snijders-Oomen Non-verbal Intelligence Tests (SON)^c^	–	2.5–40 years	Clinician	Instrument-specific training	60 min, in person	Initial kit: £1,550	SON
*The Capute Scales^*c*^*	*Communication*	*1 month−3 years*	*Parent or caregiver, clinician*	*None*	*6*–*20 min, in person*	*Initial kit: $395*	*CAPUTE Scales*
Vineland Adaptive Behavior Scale (VABS)^^p^^,^^c^^,^^s^^	Physical, Social, Communication	Up to 90 years	Parent or caregiver, clinician, teacher	None	20–45 min; in person, *via* mail, or electronic	Initial kit with 1 year license: $295	Pearson Assessments: VABS
*Visual Motor Integration Test (Beery-Buktenica)^*c*^*	–	*2–100 years*	*Clinician*	*Degree or formal training*	*10 min, in person*	*Initial kit: $168*	*Pearson Assessments: Beery-Buktenica*
Wechsler Intelligence Scale for Children (WISC-IV)^c^	–	6–16 years	Clinician	Degree or formal training	60–90 min, in person	Initial kit: $1,400	Pearson Assessments: WISC

*Italicized instruments were included via expert opinion. PICS-p domains are represented as follows:*

p *physical health*,

c *cognitive health, ^e^emotional health*,

s *social health*.

**Table 2:** Communication.

**Instrument name**	**Additional domains**	**Suggested age range**	**Data source**	**Training for administration**	**Time and method of administration**	**Cost information**	**Website/additional info**
A Developmental Neuropsychological Assessment (NEPSY) ^c^^,^ ^s^	Cognitive, Executive Functioning	3–16 years	Self-report, clinician,	Instrument-specific training	45–180 min, in person	Initial kit: $1,023	Pearson Assessments: NEPSY
Adaptive Behaviour Assessment System (ABAS) ^p^^,^ ^c^	Cognitive, Physical, Social	Up to 89 years	Self-report, parent or caregiver, teacher	Instrument-specific training	20 min, in person or electronic	Initial kit: $456	Pearson Assessments: ABAS
Ages and Stages Questionnaires^^p^^,^^c^^,^^s^^	Cognitive, Physical, Social	1 month−5.5 years	Parent or caregiver	None	10–15 min, in person	Initial kit: $295	Ages & Stages
*Bayley Scales of Infant and Toddler Development-4 *^p^^,^^c^^,^^s^**	*Cognitive, Physical, Social*	*16 days−3.5 years*	*Clinician*	*Instrument-specific training*	*30*–*70 min, in person*	*Initial kit: $1,169*	*Pearson Assessments: Bayley*
Mullen Scales of Early Learning^p^^,^ ^c^	Physical	Up to 68 months	Self-report	Degree or formal training	15–60 min, in person	Initial kit: $1,030	Pearson Assessments: Mullen Scales
*Preschool Language Scale 4^*c*^*	–	*Up to 83 months*	*Clinician*	*Instrument-specific training*	*20*–*45 min, in person*	*Initial kit: $241*	*Pearson Assessments: PLS4*
*The Capute Scales^*c*^*	*Cognitive*	*1 month−3 years*	*Parent or caregiver, clinician*	*None*	*6*–*20 min, in person*	*Initial kit: $395*	*CAPUTE Scales*
Vineland Adaptive Behavior Scale (VABS) ^^p^^,^^c^^,^^s^^	Cognitive, Physical, Social	Up to 90 years	Parent or caregiver, clinician, teacher	None	20–45 min; in person, *via* mail, or electronic	Initial kit with 1 year license: $295	Pearson Assessments: VABS

*Italicized instruments were included via expert opinion. PICS-p domains are represented as follows:*

p *physical health*,

c *cognitive health*,

s *social health*.

**Table 3:** Executive functioning.

**Instrument Name**	**Additional domains**	**Suggested age range**	**Data source**	**Training for administration**	**Time and method of administration**	**Cost information**	**Website/additional info**
A Developmental Neuropsychological Assessment (NEPSY) ^c^^,^ ^s^	Cognitive, Communication	3–16 years	Self-report, clinician,	Instrument-specific training	45–180 min, in person	Initial kit: $1,023	Pearson Assessments: NEPSY
*ADHD Rating Scale V for Children and Adolescents^c^*	–	*5*–*17 years*	*Parent or caregiver, teacher*	*None*	*5 min; in person, by phone, via mail, or electronic*	*Initial kit: $131*	*ADHD Rating Scale*
Cambridge Neuropsychological Test Automated Battery (CANTAB) ^c^^,^ ^s^	Cognitive, Social	At least 4 years	Self-report	Instrument-specific training	Depends on modules selected, (usually >30 min) electronic	Fee not available	CANTAB
Conner's Rating Scales Revised-Short Version (CRS-R:S)^c^	–	3–18 years	Self-report, parent or caregiver, teacher	None	10–20 min, in person or *via* mail	Initial kit: $309	Pearson Assessments: Conners
*Strengths & Difficulties Questionnaires (SDQ) ^*c, s*^*	*Social*	*2–18 years*	*Self-report, parent or caregiver, clinician*	*None*	*3*–*5 min; in person, by phone, via mail, or electronic*	*Free*	*SDQ*

*Italicized instruments were included via expert opinion. PICS-p domains are represented as follows:*

c *cognitive health*,

s *social health*.

**Table 4:** Physical functioning.

**Instrument name**	**Additional domains**	**Suggested age range**	**Data source**	**Training for administration**	**Time and method of administration**	**Cost information**	**Website/additional info**
36-Item Short Form Survey (SF-36)^p^^,^ ^e^^,^ ^s^	Social, Mental Health	At least 14 years	Self-report, clinician	None	10 min; in person, by phone, *via* mail, or electronic	Free	SF-36
Adaptive Behaviour Assessment System (ABAS)^p^^,^ ^c^	Cognitive, Social, Communication	Up to 89 years	Self-report, parent or caregiver, teacher	Instrument-specific training	20 min, in person or electronic	Initial kit: $456	Pearson Assessments: ABAS
Ages and Stages Questionnaires^^p^^,^^c^^,^^s^^	Cognitive, Social, Communication	1 month−5.5 years	Parent or caregiver	None	10–15 min, in person	Initial kit: $295	Ages & Stages
*Alberta Infant Motor Scale (AIMS)^*p*^*	–	*Up to 18 months*	*Clinician*	*Instrument-specific training*	*20*–*30 min, in person*	*Fee not available*	–
*Bayley Scales of Infant and Toddler Development-4*^p^^,^^c^^,^^s^**	*Cognitive, Social, Communication*	*16 days−3.5 years*	*Clinician*	*Instrument-specific training*	*30*–*70 min, in person*	*Initial kit: $1,169*	*Pearson Assessments: Bayley*
Child Health Questionnaire (CHQ)^p, c, e, s^	Cognitive, Social, Family	5–18 years	Self-report, parent or caregiver	None	5–15 min; in person, *via* mail, or electronic	Fee not available	CHQ
*Caregiver Priorities and Child Health Index of Life with Disabilities (CPCHILD)^*p, c, e, s*^*	*Cognitive, Social, Family*	*5–18 years*	*Parent or caregiver*	*None*	*20*–*40 min, in person*	*Free*	*CPCHILD*
*CHOP Infant Test of Neurologic Disorders (CHOP-INTEND)^*p*^*	–	*Up to 3 years*	*Clinician*	*Instrument-specific training*	*<20 min, in person*	*Free*	CHOP-INTEND*, designed for children with SMA and other neuromuscular disorders*
Denver Developmental Screening Test II^^p^^,^^c^^,^^s^^	Cognitive, Social	Up to 6 years	Clinician	Instrument-specific training	10–30 min, in person	Fee not available	Denver II
*Developmental Profile 3 (DP3) *^p^^,^^c^^,^^s^**	*Cognitive, Social*	*Up to 13 years*	*Self-report, parent or caregiver*	*Instrument-specific training*	*20*–*40 min, in person or by phone*	*Initial kit: $125*	*DP3*
Functional Independence Measures (FIM, WeeFIM) ^p^^,^ ^c^	Cognitive, Feeding	6 months−21 years	Self-report, parent or caregiver	Instrument-specific training	15 min; in person, *via* mail, or by phone	Licensing: $2,200–4,100	FIM/WeeFIM
Functional Status II-R (FSII-R)^^p^^,^^c^^,^^s^^	Cognitive, Social, Feeding	Up to 16 years	Parent or caregiver	None	15 min, in person	Free	–
Functional Status Scale (FSS)^p^^,^ ^c^	Cognitive, Feeding	Up to 18 years	Clinician	None	<5 min, in person or chart review	Free	FSS
Gait Outcomes Assessment List (GOAL)^p^	–	5–18 years	Self-report, parent or caregiver	None	20–30 min, in person or electronic	Free for non-commercial use (including research)	GOAL
*Glasgow Outcome Scale-Pediatrics (GOSE-Peds)^*p, c, e, s*^*	*Cognitive, Social, Family*	*Up to 16 years*	*Self-report, parent or caregiver, clinician*	*None*	*15 min, in person or via phone*	*Free*	–
*Gross Motor Function Classification System^*p*^*	–	*2–18 years*	*Self-report, parent or caregiver, clinician*	*None*	*5 min, in person, via phone, chart review*	*Free for personal, non-commercial use*	*GMFCS-E&R*
*Hammersmith Infant Neurological Examination^*p*^*	*Feeding*	*3 months−1 year*	*Clinician*	*Degree or formal training*	*10*–*15 min, in person*	*Free*	*HINE*
Heath State Utility Index (HUI)^^p^^,^^c^^,^^s^^	Cognitive, Social	5–100 years	Self-report, parent or caregiver, clinician, family member	Instrument-specific training	3–10 min; in person, by phone, or electronic	Free for information available in the literature, additional licensing: $5,000	HUI
King's Outcome Scale for Childhood Head Injury (KOSCHI)^^p^^,^^c^^,^^s^^	Cognitive, Social	Up to 16 years	Clinician	None	Variable timing, chart review	Free	KOSCHI
Lansky's Play Performance Scale for Children^p^	–	1–16 years	Parent or caregiver	None	<5 min, in person	Free	LPPSC, designed for pediatric cancer patients
*Motor Function Measure (MFM)^*p*^*	–	*6*–*60 years*	*Clinician*	*Degree or formal training*	*30*–*50 min, in person*	*Free*	*MFM*
Mullen Scales of Early Learning^p^^,^ ^c^	Communication	Up to 68 months	Self-report	Degree or formal training	15–60 min, in person	Initial kit: $1,030	Pearson Assessments: Mullen Scales
Peabody Developmental Motor Scales Assessment (PMDS-2)^p^	–	Up to 5 years	Clinician	Degree or formal training	45–60 min, in person	Initial kit: $585	Pearson Assessments: PMDS-2
Pediatric Evaluation of Disability Inventory - Computer Adaptive Test (PEDICAT) ^^p^^,^^c^^,^^s^^	Cognitive, Social, Feeding	Up to 20 years	Self-report, parent or caregiver, clinician	None	10–30 min, electronic	$2 per administration	Pearson Assessments: PEDICAT
Pediatric Overall Performance Category (POPC)^p^^,^ ^c^	Cognitive	Up to 21 years	Parent or caregiver, clinician	None	5–10 min, in person or chart review	Free	–
Pediatric Quality of Life Inventory (PedsQL)^p, c, e, s^	Cognitive, Social, Family	1 month−25 years	Self-report, parent or caregiver	None	4 min; in person, by phone, or *via* mail	Free for unfunded research; $1,089 for funded research	PEDSQL
Vineland Adaptive Behavior Scale (VABS)^^p^^,^^c^^,^^s^^	Cognitive, Social, Communication	Up to 90 years	Parent or caregiver, clinician, teacher	None	20–45 min; in person, *via* mail, or electronic	Initial kit with 1 year license: $295	Pearson Assessments: VABS
*Zurich Neuromotor Assessment^*p*^*	–	*5–18 years*	*Clinician*	*Instrument-specific training*	*20 min, in person*	*Fee not available*	–

*Italicized instruments were included via expert opinion. PICS-p domains are represented as follows:*

p *physical health,*

c *cognitive health*,

e *emotional health*,

s *social health*.

**Table 5:** Social skills.

**Instrument name**	**Additional domains**	**Suggested age range**	**Data source**	**Training for administration**	**Time and method of administration**	**Cost information**	**Website/additional info**
36-Item Short Form Survey (SF-36)^p^^,^ ^e^^,^ ^s^	Physical, Mental Health	At least 14 years	Self-report, clinician	None	10 min; in person, by phone, *via* mail, or electronic	Free	SF-36
Adaptive Behaviour Assessment System (ABAS) ^p^^,^ ^c^	Cognitive, Physical, Communication	Up to 89 years	Self-report, parent or caregiver, teacher	Instrument-specific training	20 min, in person or electronic	Initial kit: $456	Pearson Assessments: ABAS
Ages and Stages Questionnaires^p^^,^ ^c^^,^ ^s^	Cognitive, Physical, Communication	1 month−5.5 years	Parent or caregiver	None	10–15 min, in person	Initial kit: $295	Ages & Stages
Amsterdam Neuropsychological Tasks^c^^,^ ^s^	Cognitive	4–65 years	Self-report	None	315–415 min, electronic	1 year license: €1,600	ANT Program
*Bayley Scales of Infant and Toddler Development-4 *^p^^,^^c^^,^^s^**	*Cognitive, Physical, Communication*	*16 days−3.5 years*	*Clinician*	*Instrument-specific training*	*30*–*70 min, in person*	*Initial kit: $1,169*	*Pearson Assessments: Bayley*
*Behavior and Emotional Screening System (BASC)^*e, s*^*	*Mental Health*	*2–25 years*	*Self-report, parent or caregiver, teacher*	*None*	*10*–*85 min, in person or electronic*	*Initial kit: $453*	*BASC-3*
*Brief Infant Toddler Social Emotional Assessment (BITSEA)^*e, s*^*	*Mental Health*	*12*–*36 months*	*Parent or caregiver*	*None*	*5*–*7 min, in person or by mail*	*Free for clinical use and unfunded research*	*BITSEA*
Cambridge Neuropsychological Test Automated Battery (CANTAB) ^c^^,^ ^s^	Cognitive, Executive Functioning	At least 4 years	Self-report	Instrument-specific training	Depends on modules selected (usually >30 min) electronic	Fee not available	CANTAB
*Caregiver Priorities and Child Health Index of Life with Disabilities (CPCHILD)^*p, c, e, s*^*	*Cognitive, Physical, Family*	*5–18 years*	*Parent or caregiver*	*None*	*20*–*40 min, in person*	*Free*	*CPCHILD*
*Child Behavior Checklist^*e, s*^*	*Mental Health*	*18 months−18 years*	*Self-report, parent or caregiver, teacher*	*None*	*15*–*20 min; in person, via mail, or electronic*	*$295 for single user license*	*Child Behavior Checklist*
Child Health Questionnaire (CHQ)^p^^,^ ^c^^,^ ^e^^,^ ^s^	Cognitive, Physical, Family	5–18 years	Self-report, parent or caregiver	None	5–15 min; in person, *via* mail, or electronic	Fee not available	CHQ
Denver Developmental Screening Test II^p^^,^ ^c^^,^ ^s^	Cognitive, Physical	Up to 6 years	Clinician	Instrument-specific training	10–30 min, in person	Fee not available	Denver II
*Developmental Profile 3 (DP3)*^p^^,^^c^^,^^s^**	*Cognitive, Physical*	*Up to 13 years*	*Self-report, parent or caregiver*	*Instrument-specific training*	*20*–*40 min, in person or by phone*	*Initial kit: $125*	*DP3*
*Functional Status II-R (FSII-R)*^p^^,^^c^^,^^s^**	*Cognitive, Physical, Feeding*	*Up to 16 years*	*Parent or caregiver*	*None*	*15 min, in person*	*Free*	–
*Glasgow Outcome Scale-Pediatrics (GOSE-Peds)^*p, c, e, s*^*	*Cognitive, Physical, Family*	*Up to 16 years*	*Self-report, parent or caregiver, clinician*	*None*	*15 min, in person or via phone*	*Free*	–
Harter's Self-Perception Profile for Children and Adolescents (SPPC)^s^	–	8–18 years	Self-report, teacher	None	<15 min, in person	Free	SPPC
Heath State Utility Index (HUI)^p^^,^ ^c^^,^ ^s^	Cognitive, Physical	5–100 years	Self-report, parent or caregiver, clinician, family member	Instrument-specific training	3–10 min; in person, by phone, or electronic	Free for information available in the literature, additional licensing: $5,000	HUI
King's Outcome Scale for Childhood Head Injury (KOSCHI)^p^^,^ ^c^^,^ ^s^	Cognitive, Physical	Up to 16 years	Clinician	None	Variable timing, chart review	Free	KOSCHI
Pediatric Evaluation of Disability Inventory - Computer Adaptive Test (PEDICAT)^p^^,^ ^c^^,^ ^s^	Cognitive, Physical, Feeding	Up to 20 years	Self-report, parent or caregiver, clinician	None	10–30 min, electronic	$2 per administration	Pearson Assessments: PEDICAT
Pediatric Quality of Life Inventory (PedsQL)^p^^,^ ^c^^,^ ^e^^,^ ^s^	Cognitive, Physical, Family	1 month−25 years	Self-report, parent or caregiver	None	4 min; in person, by phone, or *via* mail	Free for unfunded research; $1,089 for funded research	PEDSQL
*Strengths & Difficulties Questionnaires (SDQ) ^*c, s*^*	*Executive Functioning*	*2–18 years*	*Self-report, parent or caregiver, clinician*	*None*	*3*–*5 min; in person, by phone, via mail, or electronic*	*Free*	*SDQ*
Vineland Adaptive Behavior Scale (VABS)^p^^,^ ^c^^,^ ^s^	Cognitive, Physical, Communication	Up to 90 years	Parent or caregiver, clinician, teacher	None	20–45 min; in person, *via* mail, or electronic	Initial kit with 1 year license: $295	Pearson Assessments: VABS

*Italicized instruments were included via expert opinion. PICS-p domains are represented as follows:*

p *physical health*,

c *cognitive health*,

e *emotional health*,

s *social health*.

**Table 6:** Feeding.

**Instrument name**	**Additional domains**	**Suggested age range**	**Data source**	**Training for administration**	**Time and method of administration**	**Cost information**	**Website/additional info**
Functional Independence Measures (FIM, WeeFIM) ^p^^,^ ^c^	Cognitive, Physical	6 months −21 years	Self-report, parent or caregiver	Instrument-specific training	15 min; in person, *via* mail, or by phone	Licensing: $2,200–4,100	FIM/WeeFIM
*Functional Status II-R (FSII-R)*^p^^,^^c^^,^^s^**	*Cognitive, Physical, Social*	*Up to 16 years*	*Parent or caregiver*	*None*	*15 min, in person*	*Free*	–
Functional Status Scale (FSS)^p^^,^ ^c^	Cognitive, Physical	Up to 18 years	Clinician	None	<5 min, in person or chart review	Free	FSS
*Hammersmith Infant Neurological Examination^*p*^*	*Physical*	*3 months−1 year*	*Clinician*	*Degree or formal training*	*10*–*15 min, in person*	*Free*	*HINE*
Pediatric Evaluation of Disability Inventory - Computer Adaptive Test (PEDICAT)^^p^^,^^c^^,^^s^^	Cognitive, Physical, Social	Up to 20 years	Self-report, parent or caregiver, clinician	None	10–30 min, electronic	$2 per administration	Pearson Assessments: Pearson Assessments: PEDICAT

*Italicized instruments were included via expert opinion. PICS-p domains are represented as follows:*

p *physical health*,

c *cognitive health*,

s *social health*.

**Table 7:** Family functioning and child quality of life.

**Instrument name**	**Additional domains**	**Suggested age range**	**Data source**	**Training for administration**	**Time and method of administration**	**Cost information**	**Website/additional info**
*Caregiver Priorities and Child Health Index of Life with Disabilities (CPCHILD) ^p^^,^^c^^,^^e^^,^^s^*	*Cognitive, Physical, Social*	*5–18 years*	*Parent or caregiver*	*None*	*20*–*40 min, in person*	*Free*	*CPCHILD*
Child Health Questionnaire (CHQ) ^p^^,^ ^c^^,^ ^e^^,^ ^s^	Cognitive, Physical, Social	5–18 years	Self-report, parent or caregiver	None	5–15 min; in person, *via* mail, or electronic	Fee not available	CHQ
Family Assessment Device (FAD)	–	At least 12 years	Self-report, parent or caregiver	None	15–20 min, in person	Free	FAD
*Glasgow Outcome Scale-Pediatrics (GOSE-Peds) ^p^^,^^c^^,^^e^^,^^s^*	*Cognitive, Physical, Social*	*Up to 16 years*	*Self-report, parent or caregiver, clinician*	*None*	*15 min, in person or via phone*	*Free*	–
Pediatric Quality of Life Inventory (PedsQL) ^p^^,^ ^c^^,^ ^e^^,^ ^s^	Cognitive, Physical, Social	1 month−25 years	Self-report, parent or caregiver	None	4 min; in person, by phone, or *via* mail	Free for unfunded research; $1,089 for funded research	PEDSQL

*Italicized instruments have been validated with children with disabilities. PICS-p domains are represented as follows:*

p *physical health*,

c *cognitive health*,

e *emotional health*,

s *social health*.

**Table 8:** Mental health (e.g., anxiety, depression, trauma).

**Instrument name**	**Additional domains**	**Suggested age range**	**Data source**	**Training for administration**	**Time and method of administration**	**Cost information**	**Website/additional info**
36-Item Short Form Survey (SF-36)^p^^,^ ^e^^,^ ^s^	Physical, Social	At least 14 years	Self-report, clinician	None	10 min; in person, by phone, *via* mail, or electronic	Free	SF-36
*Behavior and Emotional Screening System (BASC)^*e, s*^*	*Social*	*2–25 years*	*Self-report, parent or caregiver, teacher*	*None*	*10*–*85 min, in person or electronic*	*Initial kit: $453*	*BASC-3*
*Brief Infant Toddler Social Emotional Assessment (BITSEA)^*e, s*^*	*Social*	*12*–*36 months*	*Parent or caregiver*	*None*	*5*–*7 min, in person or by mail*	*Free for clinical use and unfunded research*	*BITSEA*
*Child Behavior Checklist^*e, s*^*	*Social*	*18 months−18 years*	*Self-report, parent or caregiver, teacher*	*None*	*15*–*20 min; in person, via mail, or electronic*	*$295 for single user license*	*Child Behavior Checklist*
*Child Depression Inventory^*e*^*	–	*7*–*17 years*	*Self-report, parent or caregiver*	*None*	*15 min, in person or electronic*	*Initial kit: $341*	*CDI*
*Child Post Traumatic Stress Disorder Symptoms Scale^*e*^*	–	*8*–*18 years*	*Self-report, clinician*	*None*	*10 min, in person*	*Free*	*PTSD Symptom Scale for DSM V*
Davidson Trauma Scales^e^^*^	–	*At least 18 years*	*Self-report*	*None*	*10 min, in person*	*Fee not available*	*DTS*
*Hospital Anxiety and Depression Score (HADS)^*e*^*	–	*At least 12 years*	*Self-report*	*None*	*20 min, in person*	*Fee not available*	*HADS*

*Italicized instruments were included via expert opinion. PICS-p domains are represented as follows:*

p *physical health*,

e *emotional health*,

s *social health*.

* *No known prior use in children specifically with disabilities or delay*.

**Table 9:** Sleep.

**Instrument name**	**Additional domains**	**Suggested age range**	**Data source**	**Training for administration**	**Time and method of administration**	**Cost information**	**Website/additional info**
Children's Sleep Habits Questionnaire	–	1 month−12 years	Parent or caregiver	None	15 min, in person, *via* mail	Free	CSHQ (Abbre *via* ted)

*Italicized instruments were included via expert opinion*.

Despite the fact that all instruments were used in children with disabilities, only 35.3% (*n* = 18) had any population information available for children with specific disabilities. The instruments were most commonly used to assess children with known cognitive (*n* = 35, 68.6%) or physical (*n* = 30, 58.8%) disabilities. The domains of functioning measured by instruments included cognitive functioning (*n* = 25 instruments, 49.0%), executive functioning (*n* = 5, 9.8%), communication (*n* = 8, 15.7%), physical functioning (*n* = 28, 54.9%), social skills (*n* = 22, 43.1%), feeding (*n* = 5, 9.8%), family functioning and child quality of life (*n* = 5, 9.8%), mental health (including anxiety, depression, and trauma) (*n* = 8, 15.7%), and sleep (*n* = 1, 2.0%).

A minority of instruments were specifically designed for (*n* = 20, 39.2%) or validated in (*n* = 24, 47.1%) populations of children with disabilities, including cerebral palsy, intellectual disabilities, and mobility limitations. This is in contrast to the 78.4% of instruments that had normative data available for the general population. With regard to properties of administration, the targeted biological ages for each instrument were broad, often in concordance with the skills measured by each instrument, with a median minimum age of 12 months (IQR 0-60). Sixteen instruments (31.4%) started at birth, while 16 (31.4%) recommended a chronological age of at least 5 years for administration. Most instruments used in-person assessments (*n* = 46, 90.2%), while smaller portions used electronic (*n* = 14, 27.5%), mail (*n* = 9, 17.6%), or telephone (*n* = 8, 15.7%) evaluation. Parents and clinicians were the most common informants (*n* = 25, 49.0% of instruments each), although self-report was also common (*n* = 24, 47.1%). Eight instruments (15.7%) could be completed by teachers. About half of the instruments (*n* = 27, 52.9%) did not require special training for administration. When special training was needed for administration, it was usually instrument-specific training (*n* = 20, 83.3% of instruments requiring special training). Estimated time for instrument completion varied across the 45 instruments for which data were available: 10 taking <10 min, 9 taking 10–15 min, 14 taking 15–30 min, and 12 taking >30 min.

Due to the nature of their design (e.g., instruments which consist of a battery of a tests completed directly with a child rather than parent questionnaires reporting on developmental skills), it is uncommon for instruments to be able to be used retrospectively to evaluate baseline function prior to an acute illness (*n* = 14, 27.5%). However, most instruments can be used prospectively, either in the hospital or in an outpatient clinic setting (62.0 and 98.0%, respectively). Most of the identified instruments (*n* = 47, 92.2%) can be used repeatedly over time.

In terms of accessibility, almost all instruments (*n* = 41, 80.4%) were available in languages other than English, with 64.7% additionally available in Spanish. In addition to English, the instruments were available in a median of 4 (IQR 1-13.8) languages. Instruments were largely proprietary (*n* = 33, 64.7%) and required a fee for use (*n* = 29, 56.9%). Pricing structures varied across instruments (see Tables 1–9 for details).

## Discussion

Children with pre-existing disabilities represent a significant portion of admissions to the intensive care unit. Due to their neurodevelopmental vulnerabilities, they are hypothesized to be at even greater risk than typically developing children of accruing new morbidity during episodes of critical illness. Therefore, clinical providers and researchers would be remiss to not explicitly consider this patient population when evaluating outcomes following critical illness, either on an individual or population basis. This study demonstrates both the value of and the challenges inherent in applying commonly used outcome measures to populations of children with disabilities beyond those seen when assessing developmental domains more broadly. Our findings demonstrate lack of population data for children with disabilities, difficulty with validation and administration specifically related to a child's disability, and potential for instruments to suffer from scale attenuation effects, potentially hampering the research necessary to improve critical care delivery to this patient population. Ideally, instruments which are explicitly designed for children with disabilities should be prioritized for use in research when assessing this patient population, but our data suggest that such instruments are rare.

Further, our content-area experts identified a number of commonly used measures for the assessment of delay and disability which had not been identified through scoping review, likely because they have not yet (to our knowledge) been applied to the PICU survivor population. These instruments included, as examples, a number of standard assessments of emotional functioning, overall developmental assessments, specific screeners for depression and ADHD, and intellectual assessments. While some of these additions require expertise for administration, others may easily be scored and interpreted using available guides. We hope that the addition of these instruments to our review may be a resource for future researchers.

We also acknowledge and encourage the assessment of children after PICU hospitalization *via* interdisciplinary collaborations. Outcome measurements that coincide with outpatient needs assessment can be coupled to screen and, if indicated, direct patients to appropriate therapies and treatments. As noted below, investigators may also leverage “baseline” assessments, when batteries have been utilized for pre-PICU, school-based, or therapy evaluations. This will potentially allow investigators to determine impacts of PICU hospitalizations as well as contribute to the optimization of long-term supports.

Despite the fact that the reviewed instruments have all been used in children with disabilities, populations with heterogeneous disabilities may need nuanced accommodations, and interpretations of population means may differ substantially from normative populations. The majority of instruments (>60%) did not have population data for children with disabilities, whereas nearly 80% of the instruments had population data available for the general population. A population of children with disabilities likely will not be comparable to the general population at the time of onset of critical illness, potentially limiting our ability to effectively interpret their post-illness state. For example, children with existing severe developmental delay are known to have low health-related quality of life (HRQL) scores when examined after septic shock (19), but it is unclear if the low HRQL scores are attributable to critical illness or different norms for HRLQ in a subset of children. It is known that some populations of children with chronic medical conditions, such as cerebral palsy or chronic respiratory failure, have lower baseline HRQL scores than the general population, perhaps due to the heavy representation of physical functioning in many HRQL scores (20–22). Therefore, we would recommend that researchers consider testing that can capture pre-critical illness functioning through retrospective reporting or by aligning with outpatient providers in order to use a change from baseline as a measure of impact.

Researchers should understand that while the stated administration ages for these instruments were generally within the pediatric age range, children with disabilities may not be best assessed by an instrument designed or validated in children with typical development, particularly if participation in an instrument involves a domain of comparative weakness (e.g., verbal responses required for a child without expressive speech or the demonstration of fine motor tasks in a child with cerebral palsy). Researchers should be particularly mindful in regards to disabilities that will require accommodation across a variety of assessment tools. For example, children with sensory impairments (e.g., vision impairment or hearing impairment) that limit ability to engage with testing materials or social impairments (e.g., autism spectrum disorder) that limit ability for social response to the examiner may be inappropriately interpreted if examiners do not select or modify an instrument to account for these impairments. In the assessment of intelligence, instead of using the Wechsler Intelligence Scale for Children (WISC-IV), non-verbal children may benefit from non-verbal tests of intelligence, e.g., the Snijders-Oomen Non-verbal Intelligence Tests (23) or the Leiter Scales (24). Additionally, researchers should be mindful that the reported time for administration of these instruments may be extended in situations where children or family members need accommodations for the tool. While ease of instrument administration is an important consideration for any patient or caregiver, it is especially key for families who may already be balancing care for medically fragile children and transporting them to appointments with assistive devices. It is also possible that some measures of neurodevelopmental, psychological, or child functioning may be re-contextualized for the measurement of post-PICU impacts in children with disabilities. For example, children may display hyperactivity or inattention in response to trauma exposure, which may be measured with tools used to diagnose and follow ADHD symptoms.

The risk for scale attenuation exists when instruments designed for typically developing children are used for children with disabilities. These instruments may not be sensitive in detecting deterioration for children whose baseline scores are significantly above or below (depending on instrument scoring) population norms. Additional difficulty exists in detecting changes with instruments whose design precludes evaluating baseline function retrospectively. An example of this would be an instrument that consists of a battery of tests for the patient rather than a questionnaire that a caregiver could fill out *via* recall at the time of PICU admission.

Researchers should prioritize instruments that allow for longitudinal assessment with the potential for retrospective data collection and the ability to endure retest scenarios to allow for establishment of the trajectory of recovery or decline after critical illness.

Although the larger critical care community also struggles with the challenges of obtaining pre-illness and longitudinal outcomes, this may be particularly true in children with disabilities. Many children with pre-existing disabilities appear to have functional or developmental declines from their pre-illness status following critical illness, concerning for development of a “new baseline” health status. However, this may instead reflect a slower recovery trajectory and long-term monitoring may provide insight into how support services can most appropriately be structured for this population. This is particularly important in a patient population that is vulnerable to recurrent need for hospitalization and the risk of cumulative morbidities. Investigators, however, should appreciate the potential advantage of studying post-PICU outcomes in patients with disabilities. Some children will have undergone community-based neurodevelopmental testing for the provision of educational and therapy services prior to an acute illness. This potentially creates a fortuitous opportunity to compare to a “true baseline” as well as collaborate with longitudinal providers. This is particularly important as assessment during acute illness is not likely to accurately reflect a child's optimal performance, whether they have underlying disabilities or not.

This study is limited by the fact that while the inclusion strategy was broad, the initial scoping review may not have adequately queried studies specifically focusing on children with disabilities, particularly when cared for in locations other than the intensive care unit. While patients with pre-existing disabilities may need a more in-depth or individualized testing battery, the scoping review largely focused on instruments used for screening a general PICU population. Additionally, we relied on manual identification of instruments used in this patient population, which may not have been uniformly performed by those extracting data. Some instruments which may be standardly used in educational or neurodevelopmental settings to evaluate functioning in diverse pediatric cohorts may not yet have been used frequently in the PICU population. Finally, the population of children with disabilities is in and of itself heterogeneous, making generalization more challenging. However, we attempted to balance these limitations through the inclusion of specific content area experts in development and behavioral pediatrics, pediatric rehabilitation medicine, and pediatric physical therapy, in addition to inclusion of instruments at the suggestion of the remainder of the authors.

Although this work is accompanied by a parallel development of a core outcome set, which included the participation of family members in its Delphi process, we did not specifically examine the outcome domains of families of children with disabilities. However, the literature identifies themes of child physical functioning, quality of life, and feeding/swallowing as important (25, 26). Many of the instruments presented in this manuscript address the identified child-focused outcome domains. However, they do not fully explore important outcomes such as care coordination, satisfaction with care, family finances, or parental outcomes. These are areas for potential further instrument development to ensure meaningful attention to patient- and family-centered outcomes whether children have disabilities or not, and we would encourage consideration of parent/family assessment of instrument utility in the development process. Additionally, little is known about how results of these instruments may be used to trigger educational or other forms of child or family support services. Ideally, these instruments would serve a dual purpose of allowing for monitoring of a patient's recovery from critical illness while also directing access to supportive and rehabilitative services.

In sum, our current ability to measure long-term outcomes for children with disabilities who experience critical illness is complicated by instruments which do not allow comparison to pre-PICU baseline and disability-specific administration concerns. Development of measures that are specifically designed for this population is important in an era where these children increasingly experience critical illness and repeated PICU admissions.

## Data Availability Statement

The raw data supporting the conclusions of this article will be made available by the authors, without undue reservation.

## Author Contributions

JAH and RJG conceived of and designed the project. JAH, SAS, and RJG drafted the manuscript and analyzed and interpreted data. All authors participated in data collection and critical revision of the article and approved the final version of the manuscript.

## Conflict of Interest

The authors declare that the research was conducted in the absence of any commercial or financial relationships that could be construed as a potential conflict of interest.
