# Supplementary material for: Outcome Measures Following Critical Illness in Children With Disabilities: A Scoping Review
Source: Front Pediatr. 2021 Jul 2;9:689485. doi: 10.3389/fped.2021.689485 (PMC8283563; doi:10.3389/fped.2021.689485)
Supplement: Supplementary file 1 [file Table_1.docx]

| **Pediatric Acute Lung Injury and Sepsis Investigators POST-PICU Investigators** | |
| --- | --- |
| **Steering Committee** | |
| Aline B. Maddux, MD, MSCS | University of Colorado School of Medicine, Children’s Hospital Colorado |
| Neethi Pinto, MD | Children’s Hospital of Philadelphia, Philadelphia, PA |
| Ericka Fink, MD | University of Pittsburgh Medical Center, Children's Hospital of Pittsburgh |
| R. Scott Watson, MD, MPH | University of Washington School of Medicine, Seattle Children’s Hospital |
| **Cognitive domain** | |
| Katherine Biagas, MD, FCCM, FAAP^a^ | The Renaissance School of Medicine at Stony Brook University |
| Mike Cronin, MD, MPH | University of Washington School of Medicine, Seattle Children’s Hospital |
| Andy Geneslaw, MD | Columbia University Irving Medical Center,  New York-Presbyterian Hospital, Morgan Stanley Children's Hospital |
| Julia A. Heneghan, MD | University of Minnesota Masonic Children’s Hospital |
| Michele Loi, MD | University of Colorado School of Medicine, Children’s Hospital Colorado |
| Sarah Murphy, MD | Harvard Medical School, MassGeneral Hospital for Children |
| Vinay Nadkarni, MD, MS | Children’s Hospital of Philadelphia, Philadelphia, PA |
| Katie R. Nielsen, MD MPH | University of Washington School of Medicine, Seattle Children’s Hospital |
| Steven L. Shein, MD | Case Western Reserve University, Rainbow Babies and Children's Hospital |
| Kristen Smith, MD, MS | University of Michigan School of Medicine, C.S. Mott Children's Hospital |
| Samer Abu-Sultaneh, MD | Indiana University School of Medicine, Riley Hospital for Children at Indiana University Health​ |
| Alexis Topjian, MD, MSCE | Children’s Hospital of Philadelphia, Philadelphia, PA |
| **Emotional domain** | |
| Reinis Balmaks, MD, PhD | Riga Stradins University, Children’s Clinical University Hospital |
| Jane (Lin) Di Gennaro, MD | University of Washington School of Medicine, Seattle Children’s Hospital |
| Reid W.D. Farris, MD, MS | University of Washington School of Medicine, Seattle Children’s Hospital |
| Madhura Hallman, MD, MPH | University of Alabama at Birmingham |
| Jessica Jarvis, PhD, MT-BC | University of Pittsburgh |
| Mara Leimanis, PhD | Helen DeVos Children’s Hospital, Michigan State University |
| Mary McNally, BSRT, RRT | Dartmouth-Hitchcock Medical Center, Children’s Hospital at Dartmouth-Hitchcock |
| Julie Menzies, PhD, MSc, B(Nurs), RNC | Birmingham Women’s and Children’s NHS Foundation Trust |
| Sholeen Nett, MD, PhD^a^ | Dartmouth-Hitchcock Medical Center, Children's Hospital at Dartmouth |
| Danielle Van Damme, DNP, CPNP-AC | University of Louisville,  Norton Children’s Hospital |
| Jane E. Whitney, MD, MSCE | Harvard Medical School, Boston Children’s Hospital |
| **Family functioning domain** | |
| Manzilat Akande, MD, MPH | The Children’s Hospital of Oklahoma, OU Medical Center |
| Mary Hartman, MD, MPH^a^ | Washington University in St Louis, St Louis Children’s Hospital |
| Kevin Hummel, MD | University of Utah Department of Pediatrics, Primary Children's Hospital |
| Catherine Madurski, MD | Nemours/Alfred I. duPont Hospital for Children in Wilmington, DE |
| Joseph C. Manning, PhD, MNursSci(Hons), PGCert Paed Crit Care, RN(Child) | Nottingham Children’s Hospital and Neonatology, Nottingham University Hospitals NHS Trust; Children and Young People’s Health Research (CYPHR), School of Health Sciences, University of Nottingham, Nottingham, UK |
| Kelly Michelson, MD, MPH, FCCM, FAAP | Northwestern University Feinberg School of Medicine, Ann & Robert H. Lurie Children’s Hospital of Chicago |
| AM Iqbal O’Meara, MD | Children's Hospital of Richmond, Virginia Commonwealth University |
| Elizabeth Pace, MD | Case Western Reserve University, Rainbow Babies and Children's Hospital |
| Monique Radman, MD, MAS | University of Washington School of Medicine, Seattle Children’s Hospital |
| Chani Traube, MD | Weill Cornell Medical Center |
| Mekela Whyte-Nesfield, MD | Children’s National Hospital |
| Lauren Yagiela, MD, MS | Children's Hospital of Michigan, Detroit, MI |
| **Health-related quality of life domain** | |
| Jonna Clark, MD, MA | University of Washington School of Medicine, Seattle Children’s Hospital |
| Robert J. Graham, MD | Boston Children’s Hospital, Department of Anesthesiology, Critical Care and Pain Medicine |
| J. Dean Jarvis, BSN, MBA, RN, CCRP | Dartmouth-Hitchcock Medical Center, Children’s Hospital at Dartmouth-Hitchcock |
| Elizabeth Y. Killien, MD, MPH^a^ | University of Washington School of Medicine, Seattle Children’s Hospital |
| Laura Loftis, MD | Baylor College of Medicine, Texas Children’s Hospital |
| Jennifer A Muszynski, MD, MPH | Nationwide Children’s Hospital |
| Brian Rissmiller, MD | Baylor College of Medicine, Texas Children’s Hospital |
| Marcy Singleton, ARNP | Dartmouth-Hitchcock Medical Center |
| Ben White, MD, MA | University of Utah Department of Pediatrics, Primary Children's Hospital |
| Jerry Zimmerman, MD, PhD, FCCM | University of Washington School of Medicine, Seattle Children’s Hospital |
| **Physical domain** | |
| Alicia M. Alcamo, MD, MPH | Children’s Hospital of Philadelphia, Philadelphia, PA |
| Stefanie G. Ames, MD MS | David Geffen School of Medicine at University of California- Los Angeles |
| Anoopindar Bhalla, MD, MSCI | University of Southern California, Children’s Hospital Los Angeles |
| Meredith Bone, MD, MSCI | Northwestern University Feinberg School of Medicine, Ann & Robert H. Lurie Children’s Hospital of Chicago |
| Karen Choong, MB, FRCP(C), MSc | McMaster University, McMaster Children’s Hospital |
| Jay M. Hunter, DNP, APRN, CPNP-AC, CCRN, CPN | University of California San Francisco School of Nursing and Benioff Children's Hospitals |
| Sapna R. Kudchadkar, MD, PhD | Johns Hopkins University School of Medicine |
| Peter M. Luckett, MD^a^ | Department of Pediatrics, University of Texas Southwestern Medical Center |
| Haifa Mtaweh, MD | The Hospital for Sick Children, University of Toronto |
| Andrew Prout, MD, MPH | University at Buffalo, Oishei Children's Hospital |
| Madiha Raees, MD | Children's Hospital of Pittsburgh |
| Joan S Roberts, MD | University of Washington School of Medicine, Seattle Children’s Hospital |
| Shilpa Shah, DO | Children’s Hospital Los Angeles |
| Alan G. Woodruff, MD | Wake Forest University School of Medicine, Brenner Children's Hospital |
| **Social domain** | |
| LeeAnn M. Christie, MSN, RN | Dell Children’s Medical Center |
| Sabrina Derrington, MD, MA (Bioethics), FAAP | Northwestern University Feinberg School of Medicine, Ann & Robert H. Lurie Children's Hospital of Chicago |
| Leslie A. Dervan, MD MS^a^ | University of Washington School of Medicine, Seattle Children’s Hospital |
| Idris Evans, MD, MSc | University of Pittsburgh Medical Center, Children's Hospital of Pittsburgh |
| Denise M. Goodman MD MS, FCCM | Northwestern University Feinberg School of Medicine, Ann & Robert H. Lurie Children’s Hospital of Chicago |
| Simon Li, MD, MPH | Maria Fareri Children’s Hospital,  New York Medical College |
| John C. Lin, MD | Washington University in St Louis, St Louis Children’s Hospital |
| Debbie Long, RN, PhD | Queensland Children’s Hospital, Brisbane, Australia |
| Maureen A. Madden MSN, RN, CPNP-AC, CCRN, FCCM | Rutgers Robert Wood Johnson Medical School, Bristol Myers Squibb Children's Hospital |
| Lindy Moake, APRN, MSN, PCCNP | Children’s Medical Center, Dallas, TX |
| Katherine N. Slain, DO | Case Western Reserve University, Rainbow Babies and Children's Hospital |
| Sara K VandenBranden, MD | Rush University Children’s Hospital, Chicago, IL |

Table S1. Pediatric Acute Lung Injury and Sepsis Investigators (PALISI) POST-PICU Scoping Review Investigators. ^a^Domain lead.
